# B vitamin acquisition by gut commensal bacteria

**DOI:** 10.1371/journal.ppat.1008208

**Published:** 2020-01-23

**Authors:** Emily E. Putnam, Andrew L. Goodman

**Affiliations:** Department of Microbial Pathogenesis and Microbial Sciences Institute, Yale University School of Medicine, New Haven, Connecticut, United States of America; Nanyang Technological University, SINGAPORE

The mammalian gut microbiome is one of the densest known microbial communities [1]. These microbial communities are largely composed of four major phyla (Bacteroidetes, Firmicutes, Proteobacteria, and Actinobacteria). Our understanding of the factors that shape gut microbial community composition is largely based on the “primary economy” of this ecosystem: the flow of carbon from the diet to bacterial biomass and fermentation products [2]. However, many enzymatic reactions in this primary economy depend on cofactors that are derived from vitamins, which are much less abundant but no less important. Vitamins may play a critical role in driving microbiome dynamics and thus provide new avenues for modification of the microbiome.

**Fig 1:**
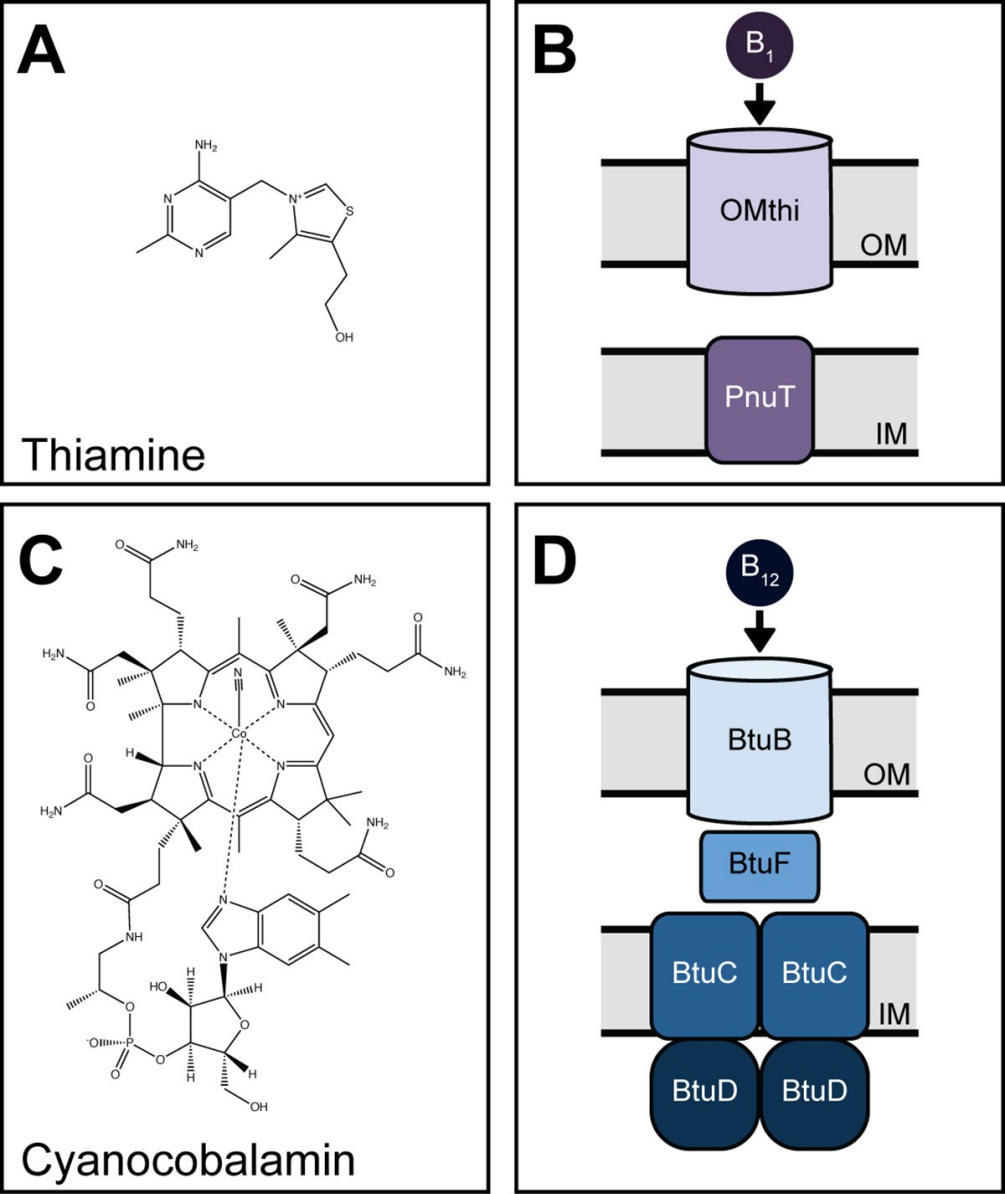
Representative B vitamins and their bacterial transporters. A and B. Thiamine structure and an example of a thiamine transport system, OMthi and PnuT, from *Bacteroides thetaiotaomicron* [3]. C and D. Cyanocobalamin and an example of a cobamide transport system, BtuBFCD, from *E*. *coli* [10]. IM, inner membrane; OM, outer membrane.

Microbes require different combinations of a variety of vitamins. These include fat-soluble vitamins, such as vitamins A, D, E, and K, and water-soluble vitamins, such as vitamin C and the B vitamins. The B vitamins are a broad category of small molecules that are important for cell metabolism but otherwise do not necessarily share structural or functional characteristics. The family of B vitamins includes thiamine (B_1_) (Fig 1A), riboflavin (B_2_), niacin (B_3_), pantothenic acid (B_5_), pyridoxine (B_6_), biotin (B_7_), folate (B_9_), and cyanocobalamin (B_12_) (Fig 1C). Cyanocobalamin belongs to the cobamide family, which includes many different vitamin B_12_–like molecules. Thiamine and cobamide provide examples of the elaborate mechanisms gut microbes use to capture vitamins.

Thiamine is required by all organisms due to its role in essential metabolic pathways, including glycolysis and the tricarboxylic acid (TCA) cycle [3]. Among gut microbes, approximately half encode the enzymes for de novo thiamine synthesis [3]. This synthesis includes production of the precursors thiazole and hydroxymethyl pyrimidine followed by the combination of these precursors into thiamine [4]. Bacteria, both within the gut and in other systems such as aquatic bacterioplankton, can acquire these precursors or mature thiamine via transport from their environment instead of or in addition to synthesis [5]. Transport of mature thiamine occurs through systems such as the ATP-binding cassette (ABC)-type transporter ThiBPQ (in Proteobacteria) and the group II energy-coupling factor (ECF) transporter ThiT (in Firmicutes) [6, 7]. Additionally, some proteins annotated as vitamin B_3_ transporters can transport thiamine, including NiaP and some Pnu transporters [8, 9]. Although the gut Bacteroidetes do not encode any previously characterized thiamine transporters, they do encode PnuT as a putative inner membrane transporter [3, 9]. Genes encoding PnuT can be in close proximity to genes for TonB-dependent outer-membrane transporters in Bacteroidetes, suggesting a functional link between TonB-dependent outer-membrane transport and PnuT-based inner-membrane transport of thiamine [4]. A heterologous expression system in *Escherichia coli* provided the first experimental support for this hypothesis [9]. Additional studies in the gut commensal *Bacteroides thetaiotaomicron* have shown that a TonB-dependent outer-membrane transporter, OMthi, is encoded near *pnuT*, and both OMthi and PnuT are involved in thiamine transport (Fig 1B) [3]. Given the wide range of strategies that bacteria encode for synthesis and transport of thiamine and the recent discovery of OMthi as a novel outer-membrane transporter of thiamine in a gut commensal, it is clear there is still much to learn about acquisition of this essential nutrient by microbes in the gut environment.

Gut microbes also exhibit varied strategies for synthesis and acquisition of cobamides. Among the cobamides, cobalamin (vitamin B_12_) is the best-known example. Cobamide-dependent enzymes can be involved in methionine synthesis, nucleotide metabolism, carbon and nitrogen metabolism, and a variety of other cell processes [10–12]. Despite these widespread functions, certain bacteria appear to lack a cobamide requirement entirely. If bacteria require cobamide, they can meet this need either through de novo synthesis, scavenging of cobamide precursors, or transport from the environment [11].

For those bacteria in the gut that are capable of de novo cobamide synthesis, the process is long and energetically costly, requiring approximately 30 enzymatic steps (reviewed in detail in [10]). Some bacteria cannot synthesize these molecules de novo but can salvage intermediates at different stages and complete synthesis. Additionally, bacteria can use alternative lower ligands in the synthesis or remodeling steps, producing the extended family of cobamides [11, 13].

Given the complexity of cobamide synthesis, it is perhaps unsurprising that many microbes encode machinery to acquire this molecule from their environment, either instead of or in addition to encoding the synthesis pathway. Over half of gut microbes are predicted to encode cobamide transporters, and vitamin B_12_ transport is an important fitness determinant for *B*. *thetaiotaomicron* in the gut environment [14, 15]. Much of what is known about vitamin B_12_ transport comes from studies of *E*. *coli*, in which an outer-membrane β-barrel protein, BtuB, brings vitamin B_12_ across the outer membrane [10]. Vitamin B_12_ then binds to the periplasmic protein BtuF, after which B_12_ crosses the inner membrane via the ABC-type transporter BtuCD (Fig 1D) [10].

While homologs of the Btu system are found across many gut bacteria, it seems unlikely that all gut bacteria use this system as observed in *E*. *coli*. For example, many gram-positive species in the gut encode *btuFCD* homologs without *btuB*, suggesting that at least some of these species use variations of the known vitamin B_12_ acquisition machinery [14, 16]. The gram-positive microbe *Lactobacillus delbrueckii* encodes an ECF-type transporter instead of an ABC-type transporter for cobamide transport [17]. Gram-negative bacteria also have variability in their transport machinery. In the soil microbe *Thiobacillus denitrificans*, the BtuM transporter not only brings cobamides across the inner membrane but can also decyanate vitamin B_12_ prior to transport [18]. Notably, *B*. *thetaiotaomicron* encodes three paralogs of *btuB* and two of *btuFCD* rather than the single set of *btuBFCD* genes observed in *E*. *coli*; other gut Bacteroidetes may encode between one and four *btuB* genes [14]. However, approximately one-quarter of the gut bacteria with a predicted cobamide requirement have no identified synthesis or transport machinery, indicating that we still have much to learn about how gut bacteria make and acquire cobamides [14].

The presence of multiple copies of cobamide transport genes in *B*. *thetaiotaomicron* and other gut bacteria presents the question of why these paralogs are advantageous to these microbes. One explanation is that these proteins have distinct functions or respond to distinct signals. Since vitamin B_12_ is a minority of the total cobamides present in the gut, variations of the transport machinery might be involved in acquisition of different types of cobamides or precursors [13, 14]. In *B*. *thetaiotaomicron*, the fitness of genetically engineered strains that encode only one of the three *btuB* alleles is dependent on the specific combination of cobamide provided and *btuB* allele [14]. This suggests that different transporters may have distinct efficiencies for transport of different cobamides and the ability to utilize different cobamides may provide a fitness advantage in the gut environment [14]. While it is not yet clear how utilization of different cobamides impacts gut species beyond *B*. *thetaiotaomicron*, many human gut *Bacteroides* encode multiple copies of cobamide transporters. The capacity to utilize different cobamides may thus influence many species, as well as the interactions between these species within the gut [19].

*B*. *thetaiotaomicron* also encodes vitamin B_12_ acquisition machinery that is not present in *E*. *coli*. A recent study characterized BtuG2, a surface-associated β-propeller that interacts with BtuB to aid vitamin B_12_ transport [20]. This protein binds vitamin B_12_ with femtomolar affinity and is capable of stripping vitamin B_12_ from the human B_12_–binding protein intrinsic factor in vitro [20]. Because humans absorb vitamin B_12_ predominantly in the small intestine and *B*. *thetaiotaomicron* and other gut commensals are found predominantly in the large intestine, vitamin B_12_ piracy from intrinsic factor by microbes is unlikely to impact vitamin B_12_ availability for the host in most cases. However, in some disease states such as small-intestinal bacterial overgrowth, this may present a link between microbial and host vitamin acquisition [20]. Homologs of *btuG* are present in all sequenced gut Bacteroidetes, suggesting that BtuG is important for cobamide acquisition among these bacteria [20].

In addition to thiamine and cobamide, gut bacteria may require other B vitamins, including riboflavin, niacin, pantothenic acid, pyridoxine, biotin, and folate. Different species may encode different combinations of the pathways for biosynthesis of these vitamins [21]. Interestingly, some pairs of gut taxa appear to have complementary biosynthetic pathways. For example, many gut Bacteroidetes encode the biosynthesis for all B vitamins except cobamide, whereas gut Firmicutes often encode just the pathway for cobamide [21]. Additionally, *Akkermansia muciniphila* and *Eubacterium hallii* engage in cross-feeding in the gut, in which *A*. *muciniphila* produces 1,2-propanediol [22]. This supports the growth of *E*. *hallii*, which in turn provides *A*. *muciniphila* with cobamide [22]. It is interesting to note that this particular example of cross-feeding involves both cobamide and a nutrient source (1,2-propanediol) that may require cobamide for utilization [10]. More broadly, the composition of the gut community does not undergo dramatic changes even in the face of dietary excess or limitation of B vitamins, suggesting that cross-feeding is an important source of these vitamins in the gut microbiome [23]. Similar processes have also been experimentally demonstrated in systems beyond the gut. For example, the aquatic algae *Ostreococcus tauri* acquires vitamin B_12_ from the bacteria *Dinoroseobacter shibae*, which in turn acquires other B vitamins from *O*. *tauri* [24]. Even more broadly, approximately half of microalgae require vitamin B_12_ acquisition from their environment, likely from neighboring bacteria, suggesting that cobamide cross-feeding is widespread [25].

Taken together, these studies highlight both the importance of vitamin acquisition for microbes in the gut environment and the diversity of mechanisms that microbes use to acquire these vitamins. These mechanisms can include de novo synthesis, salvage of intermediates, and uptake of the vitamin from the environment. Different bacterial species may utilize different machinery for synthesis or transport. It is clear that new microbial strategies are still being uncovered, such as the OMthi transporter of thiamine and the BtuG protein involved in vitamin B_12_ acquisition. While characterization of individual species provides a key starting point for understanding these processes, further studies are needed to understand how vitamin acquisition shapes competition and community structure in the context of an intact microbiome.
